# Polymicrobial bloodstream infections in the neonatal intensive care unit are associated with increased mortality: a case-control study

**DOI:** 10.1186/1471-2334-14-390

**Published:** 2014-07-14

**Authors:** Mohan Pammi, Danni Zhong, Yvette Johnson, Paula Revell, James Versalovic

**Affiliations:** 1 Section of Neonatology, Department of Pediatrics, Texas Children’s Hospital & Baylor College of Medicine, 6621, Fannin, MC: WT 6-104, Houston, TX 77030, USA; 2 Section of Neonatology, Department of Pediatrics, The First Affiliated Hospital of Guangxi Medical University, Nanning, Guangxi 530021, China; 3 Department of Pathology, Texas Children’s Hospital & Baylor College of Medicine, Houston, TX 77030, USA

**Keywords:** Neonate, Infant, NICU, Sepsis, Polymicrobial, Mortality

## Abstract

**Background:**

Polymicrobial infections in adults and children are associated with increase in mortality, duration of intensive care and healthcare costs. Very few studies have characterized polymicrobial bloodstream infections in the neonatal unit. Considerable variation has been reported in incidence of polymicrobial infections and associated clinical outcomes. We characterized the risk factors and outcomes of polymicrobial bloodstream infections in our neonatal units in a tertiary hospital in North America.

**Methods:**

In a retrospective case control study design, we identified infants in the neonatal intensive care unit with positive blood cultures at Texas Children’s Hospital, over a 16-year period from January 1, 1997 to December 31, 2012. Clinical data from online databases were available from January 2009 to December 2012. For each polymicrobial bloodstream infection (case), we matched three infants with monomicrobial bloodstream infection (control) by gestational age and birth weight.

**Results:**

We identified 2007 episodes of bloodstream infections during the 16 year study period and 280 (14%) of these were polymicrobial. Coagulase-negative Staphylococcus, Enterococcus, Klebsiella and Candida were the most common microbial genera isolated from polymicrobial infections. Polymicrobial bloodstream infections were associated with more than 3-fold increase in mortality and an increase in duration of infection. Surgical intervention was a significant risk factor for polymicrobial infection.

**Conclusion:**

The frequency and increased mortality emphasizes the clinical significance of polymicrobial bloodstream infections in the neonatal intensive care unit. Clinical awareness and focused research on neonatal polymicrobial infections is urgently needed.

## Background

Polymicrobial infections increase mortality more than 2-fold in adults and children, increase length of hospital stay and healthcare costs [1,2]. Risk factors for polymicrobial infections in children and adults include the presence of a central venous catheter, administration of parenteral nutrition, gastrointestinal pathology, especially short gut syndrome, use of broad-spectrum antibiotics and immunosuppression [1,3,4].

Neonatal polymicrobial infections are less well characterized compared to those in children or adults. Increased survival of extremely premature infants at the edge of viability, dependence on catheters and parenteral nutrition (PN), and antibiotic therapy may predispose to polymicrobial infections in neonates. The frequency of neonatal polymicrobial bloodstream infections reported in clinical studies varies from 4 to 24% of all bloodstream infections [5-10]. A standard definition for neonatal polymicrobial infections is lacking and the incidence varies from study to study partly due to variability in definition, neonatal population and practices [11]. Few studies have focused on polymicrobial infections in neonates, especially on risk factors and clinical outcomes in the western world [10-12]. In a neonatal review, the mortality due to polymicrobial infections was 3-fold greater than that of monomicrobial infections (70% vs. 23%) [10]. Organisms that are commonly implicated in neonatal polymicrobial bloodstream infections are coagulase-negative staphylococcus (CONS), Candida spp., *Staphylococcus aureus* and Enterococcus spp. [1,9,13-16]. A common risk factor appears to be multi-species biofilm infections originating from indwelling medical devices, notably indwelling vascular catheters or endotracheal tubes [1,17].

Polymicrobial bloodstream infections may be defined as multiple organisms isolated during an infectious episode including those from a single blood specimen [1] or more restrictively as isolation of more than one organism from a single blood specimen only [11,12,17]. Different definitions may partly explain the varying incidence of polymicrobial bloodstream infections reported. Bizzarro et al. (Yale, 1989-2006) and Faix et al. (Ann Arbor, 1971-1986) have reported the only two studies on neonatal polymicrobial infections from North America, [10,11]. Changing epidemiology of neonatal infections dictates a need to update and understand the epidemiology of polymicrobial infections in the hospitalized infant [11]. Lack of data on polymicrobial infections from our neonatal unit and concern of significant increase in mortality, morbidity and healthcare costs due to polymicrobial infections prompted us to investigate its frequency, risk factors and clinical outcomes at Texas Children’s Hospital (TCH).

## Methods

We hypothesized that polymicrobial infections comprise > 5% of bloodstream infections in infants residing in the neonatal intensive care units, have identifiable risk factors and are associated with higher mortality and morbidity than monomicrobial infections. We tested our hypothesis by performing a single center, retrospective, matched case-control study during a 16 year study period. Our study protocol was approved by the Institutional Review Board at Baylor College of Medicine, Houston. We followed the STROBE guidelines in reporting this study [18].

### Identification of cases and controls

We identified blood culture positive infants from the clinical microbiology database, who were admitted to the neonatal intensive care units (NICUs) III and II at TCH from January 1, 1997 to December 31, 2012. Infants more than 28 days of age in the NICU at the time of the infection and those admitted from home to the NICU with positive blood cultures were also included. The data in the clinical microbiology database is entered by the microbiologist and all positive and negative cultures including those regarded as contaminants are recorded. The tertiary NICU at TCH has approximately 1500 admissions per year including inborn and outborn transferred neonates (ranged from 1338 to 1547 during the years 2009 to 2012). Very low birth weight (VLBW, birth weight < 1500 g) infant admissions ranged from 222 to 291 infants during the years 2009 to 2012.

### Definitions

Polymicrobial bloodstream infection was defined as: i) isolation of more than one organism from a single blood culture specimen and ii) isolation of more than one organism in different blood culture specimens during the same bloodstream infectious episode. We defined ‘bloodstream infection episodes’ as time-periods associated with positive blood cultures [1] and the infection episode was considered resolved when at least two subsequent blood cultures performed every 24 hrs were negative. Usually when an organism is isolated from a blood culture specimen, blood cultures are repeated every 24 hr till two blood culture specimens are negative for organisms. Our neonatal units used only aerobic blood cultures and it is not routine to perform anaerobic blood cultures for the evaluation of neonatal sepsis. Single specimen polymicrobial infections were defined as more than one organism isolated from the same blood culture specimen. We also collected data regarding duration of the infection episode from culture positivity to culture negativity. For each polymicrobial bloodstream infection (case), we selected three gestational age matched neonates with comparable birth weights with monomicrobial bloodstream infection (control) during the time period 2009 to 2012. The matching was performed by an investigator who was blinded to neonatal risk factors and outcomes. An infant who had both polymicrobial and monomicrobial infections was included in the polymicrobial infection category for evaluation of neonatal outcomes because of the possibility that even one exposure to polymicrobial infection may increase mortality or morbidity. If an infant had multiple episodes of polymicrobial infections, the first polymicrobial infection episode was analysed. Coagulase negative staphylococcal (CONS) infections, skin flora (e.g. Micrococcus spp., Gamma Streptococci) and organisms infrequent in neonates (e.g. Bacillus spp.) were considered real infections when grown from two clinical specimens and a single culture of the above organisms were deemed contaminant and not included in the analyses.

### Clinical data collection

Neonatal clinical data including demographics, risk factors and outcomes were identified by cross referencing our institution’s neonatal clinical database (from Vermont Oxford Network (VON) database), for a 4 year period from January 1, 2009 to December 31, 2012. We collected the following clinical data for the identified cases and controls: demographic data (gestational age, birth weight, sex, age at the start of the infection episode and whether inborn or outborn), parenteral nutrition (PN) administration and its duration, presence of intravenous catheters at the time of the infectious episode (percutaneously inserted central catheters and broviac catheters but excluded umbilical catheters). We excluded umbilical venous catheters because our neonatal policy is to replace the umbilical venous catheters in the first few days of life with percutaneously inserted central catheters (PICC) and hence most umbilical lines lasted only a few days. Also, most of the bloodstream infections occurred at a time when umbilical catheters were no longer in place. We also collected data on important clinical outcomes (mortality, length of hospital stay, bronchopulmonary dysplasia, patent ductus arteriosus (PDA), necrotizing enterocolitis (NEC, ≥ stage 2 by Bell’s classification), intraventricular hemorrhage (IVH), periventricular leucomalacia (PVL) and retinopathy of prematurity (ROP), intermittent positive pressure ventilation (IPPV) at 36 wks corrected gestational age (GA), continuous positive airway pressure (CPAP) at 36 wks corrected GA, high frequency oscillatory ventilation (HFOV), inhaled nitric oxide (INO) therapy, extracorporeal membrane oxygenation (ECMO), surgeries, direct hyperbilirubinemia (direct bilirubin > 2 mg/dl or > 15% of total bilirubin) congenital heart disease and congenital malformations. All outcomes were defined as per VON database definitions.

### Statistical analyses

Data were analyzed using STATA 11, Stata Corporation, Dallas, USA. Infants with polymicrobial bloodstream infections were compared with three gestational age and birth weight matched infants with monomicrobial bloodstream infections. Continuous data were analyzed for statistical significance by the Student’s *t* test and categorical data were analyzed by chi squared analyses. A p value of < 0.05 was considered significant. A logistic regression analysis was performed for binary outcomes and odds ratios with 95% confidence intervals were estimated. The outcome of mortality was analyzed adjusting for ‘any surgery’ in the logistic regression model. Polymicrobial infections were used as the outcome of the logistic regression analysis for risk factors and as a covariate when assessing for neonatal outcomes. A subgroup analysis of VLBW infants was performed for mortality and risk factors in the logistic regression model.

## Results

**Figure 1:**
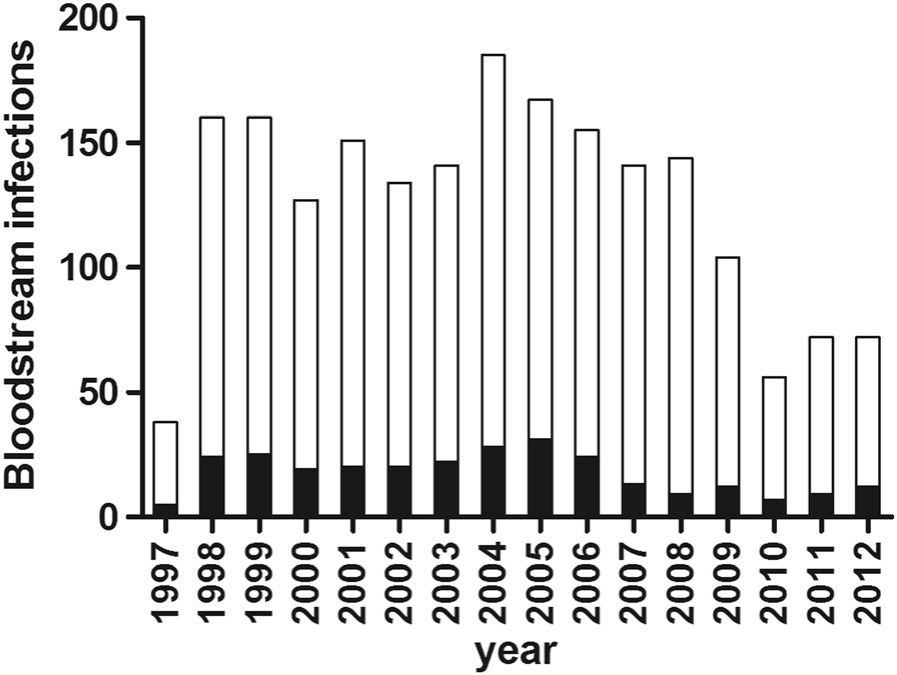
**Frequency of polymicrobial bacteremia episodes.** The frequencies of neonatal bacteremia episodes are plotted against the years from 1997 to 2012. The dark shaded portion of the bars indicate polymicrobial component of the infectious episodes, which ranged from 6.3 to 18.6% and on an average of about 14% during the 16-year study period. There were significant decrease in the number of infections and polymicrobial infections between the time epochs, 1998-2009 and 2010-2012 (p < 0.01).

We identified 2007 bacteremia episodes during a period of 16 years in patients admitted to the NICUs at TCH; 280 episodes (14%) were polymicrobial (Figure 1). The percentage of annual polymicrobial bacteremia varied during the study period ranging from 6.3 to 18.6%. Polymicrobial bacteremia identified in single blood specimens constituted on an average of 56% (range 40 to 75%) during the 16 year study period.

Microbes isolated from monomicrobial (controls) and polymicrobial infections (cases) were similar but varied in frequency (Table 1). Microbes isolated at greater frequencies in polymicrobial infections were *Candida* spp. (>3%), *Enterococcus faecalis* (>2-fold) and *Klebsiella pneumoniae* (>2-fold). The most common combinations of polymicrobial organisms were CONS and *Candida spp.* (n = 6) and CONS and *Enterococcus faecalis* (n = 3). Gram positive organisms were isolated more frequently than gram negative organisms and the proportion of gram positive to gram negative organisms were similar in both monomicrobial (gram positive 51% and gram negative 42%) and polymicrobial infections (gram positive 48% and gram negative 40%).

**Table 1:** Microbiology of monomicrobial and polymicrobial bloodstream infections

	**Monomicrobial infections data as n (%)**	**Polymicrobial infections data as n (%)**
CONS	16 (15.69)	12 (16.22)
*Staphylococcus aureus*	16 (15.69)	6 (8.11)
*Streptococcus agalactiae*	6 (5.88)	2 (2.70)
*Candida albicans, C. parapsilosis, C. glabrata*	7 (6.86)	7 (9.46)
*Escherichia coli*	18 (17.65)	5 (6.76)
*Enterococcus faecalis, E. faecium*	7 (6.86)	11 (14.86)
*Enterobacter cloacae, E. aerogenes*	8 (7.84)	5 (6.76)
*Klebsiella pneumoniae, K. oxytoca*	6 (5.88)	11 (14.86)
*Pseudomonas aeruginosa*	4 (3.92)	4 (5.41)
Bacillus spp.	4 (3.92)	1 (1.35)
*Serratia marcescens*	3 (2.94)	3 (4.05)
Others	7 (6.86)	7 (9.46)
Total	102 (100)	72 (100)

In monomicrobial bloodstream infections, 102 organisms were isolated from 102 infectious episodes. In polymicrobial bloodstream infections, 74 organisms were isolated from 34 infectious episodes. **‘**Others’ include *Streptococcus salivarius*, *Citrobacter freundii*, *Clostridium tertium* for monomicrobial infections and for polymicrobial infections; they were Lactobacillus, *Proteus mirabilis*, *Streptococcus sanguinus*, Micrococcus spp., *Acinetobacter baumanii* complex and Gamma Streptococcus. All the organisms in the ‘Others’ group was cultured at least twice from 2 different samples. Candida species, Enterococcus species and Klebsiella species were isolated at higher frequencies in polymicrobial infections than monomicrobial infections. The most common combination of polymicrobial organisms were CONS and Candida (n = 6) and CONS and *Enterococcus fecalis* (n = 3).

We compared clinical data pertaining to demographics, risk factors and clinical outcomes between infants with polymicrobial bacteremia and gestational age and birthweight matched infants with monomicrobial bacteremia (data from January 2009 to December 2012) (Table 2). We obtained neonatal data for 34 infants with polymicrobial bacteremia and 102 matched infants with monomicrobial bacteremia during that period. The demographics were similar between infants with polymicrobial compared to infants with monomicrobial bacteremia. As expected, the gestational age (30.5 vs. 30.4 wk, p = 0.90) and birth weights (1592 vs.1535 g, p = 0.77) were adequately matched between controls and cases respectively. Male sex and age at infection did not differ significantly between cases and controls.

**Table 2:** Infant demographics, risk factors and outcomes in cases and controls

	**Monomicrobial infections (n = 102), Mean [95% CI] or n (%)**	**Polymicrobial infections (n = 34), Mean [95% CI] or n (%)**	**p value**	**Odds ratio (OR) [95% CI]**
**Demographics**				
GA (wks)	30.5 [29.9, 31.1]	30.4 [29.4, 31.3]	0.90	
Bwt (g)	1592 [1493, 1692]	1535 [1365, 1706]	0.77	
Male sex, n (%)	56 (54)	17 (50)	0.65	
Age at infection (days)	40.7 [35.8, 45.5]	37.7 [30.3, 45.0]	0.75	
Inborn (%)	43 (41.2)	14 (41.2)	1.00	
**Risk factors**				
Catheter, n (%)	93 (91)	33 (97)	0.20	3.6 [0.4 to 29.1]
PN, n (%)	92 (90.2)	30 (88.2)	0.74	
PN duration (days)	49.3 [43.9, 54.8]	47.5 [40.0, 55.1]	0.86	
NEC (%)	24 (23.5)	7 (20.6)	0.72	0.8 [0.3 to 2.2]
NEC surgery (%)	18 (17.6)	6 (17.6)	1.00	1.0 [0.4 to 2.8]
Surgery other than NEC (%)	55 (53.9)	25 (73.5)	0.04*	2.4 [1.0 to 5.6]
Any surgery (%)	56 (54.9)	25 (73.5)	0.05*	2.3 [1.0 to 5.4]
Direct hyperbilirubinemia (%)	33 (32.4)	17 (50.0)	0.06	2.1 [1.0 to 4.6]
Congenital heart disease (%)	28 (27.5)	9 (26.5)	0.91	1.0 [0.4 to 2.3]
Congenital malformations (%)	31 (30.4)	15 (44.1)	0.14	1.8 [0.8 to 4.0]
**Outcomes**				
Mortality (%)	19.6	47.1	0.001 *	4.3 [1.8 to 10.2] Logistic model adjusted for surgery
Hospital stay (days)	101.7 [93.6, 109.8]	100.5 [88.1, 112.9]	0.99	
Infection duration (days)	2.1 [1.9, 2.2]	2.9 [2.5, 3.3]	0.02*	
BPD (%)	35 (34.3)	9 (26.5)	0.40	0.7 [0.3 to 1.6]
IPPV at 36wks (%)	24 (23.7)	12 (35.3)	0.19	1.8 [0.8 to 4.1]
CPAP at 36wks (%)	8 (7.8)	4 (11.8)	0.49	1.6 [0.4 to 5.6]
HFOV (%)	33 (32.4)	10 (29.4)	0.75	0.9 [0.4 to 2.0]
ECMO (%)	16 (15.7)	6 (17.6)	0.79	1.2 [0.4 to 3.2]
INO (%)	26 (25.5)	14 (41.2)	0.08	2.1 [0.9 to 4.6]
INO or HFOV or ECMO (%)	52 (50.9)	18 (52.9)	0.84	1.1 [0.5 to 2.4]
PDA (%)	13 (32.4)	33 (38.2)	0.53	1.3 [0.6 to 2.9]
ROP (%)	12 (11.8)	4 (11.8)	1.00	1.0 [0.3 to 3.3]
Severe ROP (%)	7 (6.9)	3 (8.8)	0.70	1.3 [0.3 to 5.4]
ROP surgery (%)	6 (5.9)	2 (5.9)	1.00	1.0 [0.2 to 5.2]
Severe IVH (%)	18 (17.6)	5 (14.7)	0.69	0.8 [0.3 to 2.4]
Severe IVH or PVL (%)	19 (19.6)	5 (14.7)	0.52	0.8 [0.3 to 2.2]

95% CI- 95% confidence intervals, Bwt-Birth weight, GA-gestational age, PN- parenteral nutrition, NEC- necrotizing enterocolitis, BPD-bronchopulmonary dysplasia, IPPV- Intermittent positive pressure ventilation, CPAP- continuous positive airway pressure, HFOV- High frequency oscillatory ventilation, INO- inhaled nitric oxide, ECMO- extracorporeal membrane oxygenator, PDA-patent ductus arteriosus, ROP-retinopathy of prematurity, IVH-intraventricular hemorrhage, PVL- periventricular leucomalacia, severe IVH- IVH > grade 2, severe ROP- ROP > stage 2. *p values < 0.05.

We observed a significant difference between cases and controls in terms of surgery other than for NEC (p = 0.04) and any surgery (including NEC surgery) (p = 0.05, borderline significance). We observed an increase in the presence of a central venous catheter (97 vs. 91%, p =0.20) and incidence of direct hyperbilirubinemia (50 vs. 32%, p = 0.06) both of which did not reach statistical significance. We did not observe any significant differences in the proportion of infants receiving PN, days on PN, the incidence of NEC, surgery for NEC, congenital heart disease or congenital malformations between cases and controls.

We noted a significant difference in associated mortality between controls and cases (47 vs. 20%), odds ratio adjusted for ‘any surgery’, 4.3 [95% CI, 1.8 to 10.2] (p = 0.001). A significant increase in the duration of infection (culture positivity) was noted between cases and controls (2.91 vs. 2.07 days, p = 0.02). No significant differences were observed in length of hospital stay, BPD, IPPV at 36 corrected wks, CPAP at 36 corrected wks, HFOV, ECMO, PDA, severe IVH (IVH > grade 2), PVL, severe IVH or PVL, ROP or severe ROP (ROP > stage 2) (p > 0.05). An increase in INO therapy in cases were noted (41 vs. 25%, p = 0.08) but was not statistically significant.

In a subgroup analysis of VLBW infants, polymicrobial infections compared to monomicrobial infections, had a significantly higher mortality (OR 6.4 [95% CI 1.3 to 30.3], longer duration of infection (OR 1.4 [95% CI 1.04 to 1.8] and higher incidence of congenital malformations (OR 17.7 [95% CI, 2.1 to 148.7).

## Discussion

We performed a case-control study of neonatal polymicrobial infections in a tertiary hospital in North America, and identified polymicrobial bloodstream infections in nearly 14% of bloodstream infections. We also probed the electronic neonatal clinical database over a four year period from January 2009 to December 2012 for relevant clinical data including risk factors and outcomes. We observed that polymicrobial bloodstream infections were associated with an increased mortality (>3-fold) and increased duration of infection compared to monomicrobial bloodstream infections. Surgical intervention excluding NEC surgery was a significant risk factor for neonatal polymicrobial infections.

The annual frequency of polymicrobial bloodstream infections in our study was approximately 14% over a 16 year study period. The frequency of neonatal polymicrobial infections reported in literature is variable, ranging from 3.9 to 25% [10-12,17,19-21]. Contaminated multi-dose lipid emulsions were responsible for the high frequency (25%) of polymicrobial infections in one study [20]. In most studies that report neonatal infections, the isolation of multiple organisms is often not discussed. The higher frequency of polymicrobial infections in our study compared to other reports in literature may be due to two reasons. First is the varying definition of polymicrobial infections in the different studies. We defined polymicrobial bloodstream infections as multiple organisms isolated during an infectious episode including those from a single blood specimen similar to Sutter et al., as we believe that this is a true reflection of a polymicrobial infection [1]. Other studies defined polymicrobial infections as multiple pathogens isolated from a single blood specimen, which may account for the lower incidence of polymicrobial infections [11,12,17]. Using the latter definition the annual frequency of polymicrobial bloodstream infections in our study would be approximately 7.8%, comparable to the 10% frequency reported in North American by Bizzarro et al. [11]. Secondly, increased incidence of polymicrobial bloodstream infections in our study may also relate to the large percentage of medically complex infants referred to our NICU including infants requiring surgical interventions, complex congenital heart disease, ECMO, abdominal wall defects, short gut syndrome and other congenital anomalies. This complex group of infants often requires indwelling vascular catheters for parenteral nutrition or medications for extended periods of time. The annual frequency of polymicrobial bacteremia in the neonatal intensive care unit in our study varied from 6.3 to 18.6% of all bacteremia during the 16-year study period. This annual variation may be in part due to expansion of the NICU, changes in the case-mix and referral patterns, nursing policy changes or implementation of catheter care bundles. There were significant decrease in the number of infections and polymicrobial infections between the time epochs, 1998-2009 and 2010-2012 (p < 0.01). We did not note any significant differences in the number of admissions of VLBW and ELBW infants. A dedicated vascular access team was formed in June 2009 followed by better implementation of catheter care bundles in June 2009, which along with increased compliance with hand hygiene and other infection control measures may have contributed to the decrease in infections.

In our study, cases and controls had similar demographics, a mean gestational age of approximately 30 weeks, birth weight of approximately 1500 g, similar male to female ratio and similar percentage being inborn. We focused on risk factors reported for polymicrobial infections in existing literature. The average age at the onset of the infections was 41 days in controls and 38 days in cases and hence all infections were in the late onset sepsis category and mostly occurred beyond the first 28 days of life. Almost all of the infections were late-onset infections and hence we did not collect data on risk factors for early onset sepsis such as maternal prolonged rupture of membranes, group B streptococcus colonization, urinary tract infection or chorioamnionitis. Subgroups of infections in neonates less than 28 days of age or those that were admitted from home were too small for meaningful comparisons. We noted a significant association of polymicrobial infections with surgery other than NEC and a trend towards association with direct jaundice. Cases had a higher incidence of central venous catheter compared to controls (97 vs. 91%, p = 0.20) but this association was not statistically significant.

In our case-control study, we observed more than 3-fold increase in associated mortality in neonates with polymicrobial bacteremia, but the retrospective and database oriented nature of our study precludes any conclusions of causality. Mortality due to polymicrobial infections has been reported to be at least 2-fold more than that of monomicrobial infections both in adults and children [1,2]. Faix et al. reported that mortality due to polymicrobial infections in neonates is increased almost 3-fold in his study of 15 cases of polymicrobial infections in 1971-86 [10]. More recent studies by Bizzarro et al. (study period 1989-2006), Tsai et al (study period 2004 to 2011) and Gupta et al. (1 year period in early 2000) did not report an increase in attributable mortality to polymicrobial infections in their studies [11,12,17]. The variability in mortality rates reported by different studies on polymicrobial infections may be multifactorial including differences in study design, variable definitions of polymicrobial infections, patient population, study periods, virulence of the organisms isolated or other unidentified factors [11]. The mechanisms for increased mortality in polymicrobial infections are not clear. Similar increases in mortality are observed in animal models of systemic and local polymicrobial infections [22-25]. We have observed increased catheter infection and systemic dissemination in a polymicrobial biofilm catheter infection model compared to monomicrobial infection [11]. The increased mortality may also arise from inherent host vulnerability that causes the infection in the first place (e.g. prematurity, short gut) or facilitation of one infection by the other (synergism) [26]. Polymicrobial interactions in polymicrobial environments such as a mixed-species biofilms on catheters may induce a host of synergistic mechanisms including quorum sensing, induction of virulence among others that may contribute to enhanced mortality or morbidity of the host [12].

We also noted an increase in the duration of infection in cases compared to controls, which may be due to increased host susceptibility or a synergistic effect of the polymicrobial infection. We did not observe any differences in length of hospital stay or other clinically relevant neonatal outcomes such as BPD, IPPV or CPAP at 36 corrected weeks, HFOV, ECMO, ROP, NEC or IVH or PVL. We observed an increase in the use of INO therapy in cases compared to controls, which was not statistically significant.

The organisms isolated in our study were mostly similar in both monomicrobial and polymicrobial bloodstream infections, similar in antibiotic susceptibility patterns and comparable to those organisms from polymicrobial infections reported in literature. The most common organisms isolated in both monomicrobial and polymicrobial infections were CONS, *Staphylococcus aureus*, *Escherichia coli*, *Enterococcus* species, *Klebsiella* spp. and *Candida* spp. Infections due to *Candida* spp., *Enterococcus* species and *Klebsiella* spp. increased in frequency in cases compared to controls. The most frequent polymicrobial combinations were CONS with *Candida* spp. and other organisms with *Enterococcus faecalis*, similar to reported literature [1,14,27]. Studies from Asia, by Tsai et al. and Gupta et al. report a high incidence of Gram negative infections (approximately 60%) in the polymicrobial group [12,17]. In the North American study by Bizarro et al., polymicrobial infections were preponderantly caused by Gram positive organisms (77%) similar to our study [11]. Geographic variations in organisms isolated from neonatal polymicrobial infections and their antibiotic susceptibility patterns may partly explain the variation in mortality and morbidity across the world due to polymicrobial infections. In our study we did not discern any differences in the treatment regimens used to treat polymicrobial infections and monomicrobial infections that could explain the differences in mortality. Polymicrobial infections were treated adequately for all the organisms isolated and duration of therapy was consistent with our written neonatal guidelines.

Limitations of our study include being a retrospective, observational, case-control study. Bloodstream episodes were identified from the clinical microbiology database and hence the clinical data associated with the infection or their severity were not available. The clinical risk factors and outcomes were identified from the clinical database, which was available only for a 4 year period. Being a retrospective study, it is difficult to assign a causal relationship to the increased mortality associated with polymicrobial infections in our study. We did not assess long term developmental or growth outcomes as longitudinal assessment data were not available.

The human microbiome project and other microbiome studies emphasize the polymicrobial nature of organism communities in the human body [28,29]. The availability of molecular culture-independent methods for detection of sepsis may increase the identification of polymicrobial infections [30]. Guidelines or recommendations for therapeutic and preventive strategies against polymicrobial infections do not exist. Prolonged therapy with antibiotics targeting all organisms isolated in a polymicrobial infection and removing infected catheters remain the mainstay in therapy. Defining the epidemiology and clinical impact of polymicrobial infections may be the first step towards delineating optimal therapy and clinical outcomes. Preventive strategies should emphasize catheter care bundles that decrease central line associated bloodstream infections. Focused research is necessary to prevent and treat polymicrobial infections to improve clinical outcomes in the vulnerable neonate.

## Conclusions

The main aim of the study was to investigate the frequency of polymicrobial infections in our tertiary neonatal intensive care unit, understand its impact on neonatal outcomes and promote research in the management of these infections. Polymicrobial bacteremia is common in neonates and comprises nearly 14% of all infectious episodes. The most common organisms in neonatal polymicrobial infections are CONS, *S. aureus*, *Enterococcus* species, *E. coli,* and *Candida* species. Surgical intervention is a significant risk factor for neonatal polymicrobial infections. We observed a more than 3-fold increase in mortality in infants with polymicrobial bacteremia and a significant increase in duration of the bacteremia. Research focused to prevent and to improve clinical outcomes in neonatal polymicrobial infections is urgently needed.

## Abbreviations

TCH: Texas Children’s Hospital; VON: Vermont Oxford Network; CONS: Coagulase negative Staphylococci; ELBW: Extremely low birth weight; VLBW: Very low birth weight; NICU: Neonatal Intensive Care Unit; PDA: Patent ductus arteriosus; BPD: Bronchopulmonary dysplasia; PVL: Periventricular leucomalacia; NEC: Necrotizing enterocolitis; TPN: Total parenteral nutrition; ROP: Retinopathy of prematurity; IVH: Intraventricular hemorrhage; IPPV: Intermittent positive pressure ventilation; CPAP: Continuous positive airway pressure; HFOV: High frequency oscillatory ventilation; INO: Inhaled nitric oxide.

## Competing interests

None of the authors have any financial interests to disclose or competing interest.

## Authors’ contributions

We state that all the authors satisfy the requirements of author as laid down in ‘Instructions to the Authors’. **MP** conceived the project, participated in the design, data acquisition, analysis and interpretation, drafted the initial manuscript and approved the final manuscript as submitted. **DZ** participated in the acquisition of microbiology data and tabulating the data. **YJ** for clinical data acquisition and revision of the manuscript. **PR** participated in microbiology data acquisition, analysis and revision of the manuscript. **JV** for critical intellectual input and revision of the manuscript. All authors read and approved the final manuscript.

## Pre-publication history

The pre-publication history for this paper can be accessed here:

http://www.biomedcentral.com/1471-2334/14/390/prepub
